# 2-[Bis(3,5-dimethyl­phen­yl)­phosphor­yl]­propan-2-ol hemihydrate

**DOI:** 10.1107/S1600536808016681

**Published:** 2008-06-07

**Authors:** Qing-Yan Chu, Shan Liu, Yuan-Yuan Liu, Wei Chen, Hong-Jun Zhu

**Affiliations:** a Department of Applied Chemistry, College of Science, Nanjing University of Technology, Nanjing 210009, People’s Republic of China

## Abstract

In the organic mol­ecule of the title compound, C_19_H_25_O_2_P·0.5H_2_O, the benzene rings are oriented at a dihedral angle of 54.04 (3)°. Intra­molecular C—H⋯O hydrogen bonds result in the formation of two five-membered planar rings, which are oriented with respect to the adjacent benzene rings at dihedral angles of 2.66 (3) and 2.79 (3)°. In the crystal structure, inter­molecular O—H⋯O hydrogen bonds link the mol­ecules. The water oxygen atom lies on a twofold rotation axis.

## Related literature

For related literature, see: Takao & Kazuhiko (1997 ▶). For bond-length data, see: Allen *et al.* (1987 ▶).
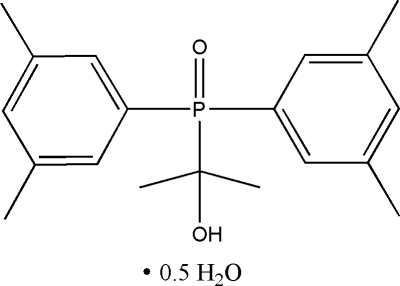


## Experimental

### 

#### Crystal data

C_19_H_25_O_2_P·0.5H_2_O
*M* *_r_* = 324.87
Monoclinic, 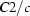

*a* = 30.129 (6) Å
*b* = 6.2830 (13) Å
*c* = 20.192 (4) Å
β = 106.76 (3)°
*V* = 3660.1 (14) Å^3^
*Z* = 8
Mo *K*α radiation
μ = 0.16 mm^−1^
*T* = 298 (2) K
0.20 × 0.10 × 0.10 mm

#### Data collection

Enraf–Nonius CAD-4 diffractometer
Absorption correction: ψ scan (North *et al.*, 1968 ▶) *T*_min_ = 0.969, *T*_max_ = 0.984
6573 measured reflections
3298 independent reflections
1904 reflections with *I* > 2σ(*I*)
*R*_int_ = 0.049
3 standard reflections frequency: 120 min intensity decay: none

#### Refinement

*R*[*F* ^2^ > 2σ(*F* ^2^)] = 0.060
*wR*(*F* ^2^) = 0.205
*S* = 1.03
3298 reflections
206 parameters
H-atom parameters constrained
Δρ_max_ = 0.49 e Å^−3^
Δρ_min_ = −0.55 e Å^−3^

### 

Data collection: *CAD-4 Software* (Enraf–Nonius, 1985 ▶); cell refinement: *CAD-4 Software*; data reduction: *XCAD4* (Harms & Wocadlo, 1995 ▶); program(s) used to solve structure: *SHELXS97* (Sheldrick, 2008 ▶); program(s) used to refine structure: *SHELXL97* (Sheldrick, 2008 ▶); molecular graphics: *SHELXTL* (Sheldrick, 2008 ▶); software used to prepare material for publication: *SHELXTL*.

## Supplementary Material

Crystal structure: contains datablocks I, Ils. DOI: 10.1107/S1600536808016681/hk2469sup1.cif

Structure factors: contains datablocks I. DOI: 10.1107/S1600536808016681/hk2469Isup2.hkl

Additional supplementary materials:  crystallographic information; 3D view; checkCIF report

## Figures and Tables

**Table 1:** Hydrogen-bond geometry (Å, °)

*D*—H⋯*A*	*D*—H	H⋯*A*	*D*⋯*A*	*D*—H⋯*A*
O*W*—H*WA*⋯O2^i^	0.85	2.24	2.811 (3)	124
O1—H1*A*⋯O2^ii^	0.82	1.96	2.775 (4)	178
C3—H3*A*⋯O2	0.93	2.47	2.928 (5)	110
C11—H11*A*⋯O2	0.93	2.49	2.939 (5)	110

Symmetry codes: (i) 
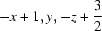
; (ii) 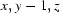.

**Fig. 1.**
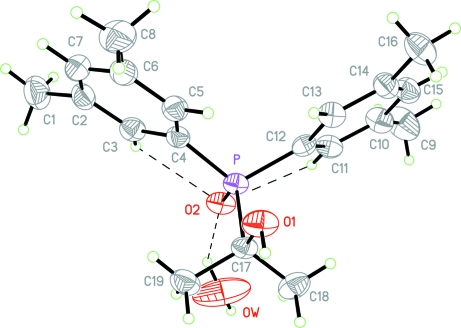
The molecular structure of the title molecule, with the atom-numbering scheme. Displacement ellipsoids are drawn at the 40% probability level. Hydrogen bonds are shown as dashed lines.

**Fig. 2.**
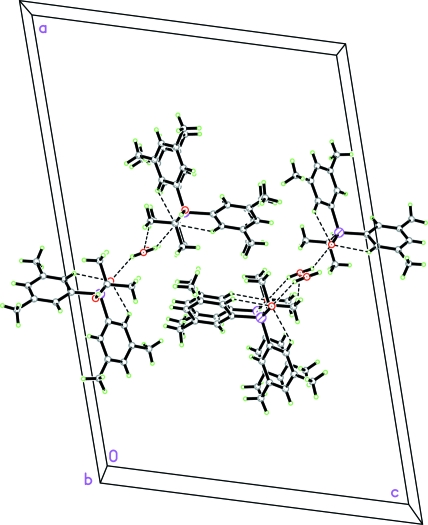
A packing diagram of (I). Hydrogen bonds are shown as dashed lines.

## supplementary crystallographic information

### Comment

2-(Bis(3,5-dimethylphenyl)phosphoryl)propan-2-ol was first synthesized by the nucleophillic addition of acetone with di(3,5-dimethylphenyl)phosphine oxide at room temperature. We report herein its crystal structure.

In the molecule of the title compound, (I), (Fig. 1) the bond lengths (Allen *et al.*,  1987) and angles are within normal ranges. Rings A (C1-C6) and B (C10-C15) are, of course, planar, and the dihedral angle between them is A/B = 54.04 (3)°. The intramolecular C-H···O hydrogen bonds (Table 1) result in the formation of two five-membered planar rings: C (C3/C4/P/O2/H3A) and D (C11/C12/P/O2/H11A). The dihedral angles between the adjacent rings are A/C = 2.66 (3)° and B/D = 2.79 (3)°. So, rings A, C and B, D are nearly coplanar. The coplanar ring systems are oriented at a dihedral angle of 54.70 (3)°.

In the crystal structure, intermolecular O-H···O hydrogen bonds (Table 1) link the molecules (Fig. 2), in which they may be effective in the stabilization of the structure.

### Experimental

The title compound, (I) was synthesized by the reaction of di(3,5-dimethyl- phenyl)phosphine oxide (0.20 g, 0.70 mmol) (Takao & Kazuhiko, 1997) and acetone (25 ml). Crystals suitable for X-ray analysis were obtained by dissolving (I) in acetone and evaporating the solvent slowly at room temperature for about 7 d.

### Refinement

H atoms were positioned geometrically, with O-H = 0.85 Å (for H_2_O) and 0.82 Å (for OH) and C-H = 0.93 and 0.96 Å for aromatic and methyl H and constrained to ride on their parent atoms with U_iso_(H) = xU_eq_(C,O), where x = 1.5 for OH and methyl H and x = 1.2 for all other H atoms.

### Crystal data

**Table:**

C_19_H_25_O_2_P·0.5H_2_O	*F*_000_ = 1396
*M**_r_* = 324.87	*D*_x_ = 1.179 Mg m^−^^3^
Monoclinic, *C*2/*c*	Mo *K*α radiation λ = 0.71073 Å
Hall symbol: -C 2yc	Cell parameters from 25 reflections
*a* = 30.129 (6) Å	θ = 9–12º
*b* = 6.2830 (13) Å	µ = 0.16 mm^−^^1^
*c* = 20.192 (4) Å	*T* = 298 (2) K
β = 106.76 (3)º	Needle, colorless
*V* = 3660.1 (14) Å^3^	0.20 × 0.10 × 0.10 mm
*Z* = 8	

### Data collection

**Table:**

Enraf–Nonius CAD-4 diffractometer	*R*_int_ = 0.049
Radiation source: fine-focus sealed tube	θ_max_ = 25.2º
Monochromator: graphite	θ_min_ = 1.4º
*T* = 298(2) K	*h* = −36→34
ω/2θ scans	*k* = 0→7
Absorption correction: ψ scan(North *et al.*, 1968)	*l* = 0→24
*T*_min_ = 0.969, *T*_max_ = 0.984	3 standard reflections
6573 measured reflections	every 120 min
3298 independent reflections	intensity decay: none
1904 reflections with *I* > 2σ(*I*)	

### Refinement

**Table:**

Refinement on *F*^2^	Secondary atom site location: difference Fourier map
Least-squares matrix: full	Hydrogen site location: inferred from neighbouring sites
*R*[*F*^2^ > 2σ(*F*^2^)] = 0.060	H-atom parameters constrained
*wR*(*F*^2^) = 0.205	*w* = 1/[σ^2^(*F*_o_^2^) + (0.1*P*)^2^] where *P* = (*F*_o_^2^ + 2*F*_c_^2^)/3
*S* = 1.03	(Δ/σ)_max_ = 0.001
3298 reflections	Δρ_max_ = 0.49 e Å^−^^3^
206 parameters	Δρ_min_ = −0.55 e Å^−^^3^
Primary atom site location: structure-invariant direct methods	Extinction correction: none

### Special details

**Table:**

Geometry. All e.s.d.'s (except the e.s.d. in the dihedral angle between two l.s. planes) are estimated using the full covariance matrix. The cell e.s.d.'s are taken into account individually in the estimation of e.s.d.'s in distances, angles and torsion angles; correlations between e.s.d.'s in cell parameters are only used when they are defined by crystal symmetry. An approximate (isotropic) treatment of cell e.s.d.'s is used for estimating e.s.d.'s involving l.s. planes.
Refinement. Refinement of F^2^ against ALL reflections. The weighted R-factor wR and goodness of fit S are based on F^2^, conventional R-factors R are based on F, with F set to zero for negative F^2^. The threshold expression of F^2^ > 2sigma(F^2^) is used only for calculating R-factors(gt) etc. and is not relevant to the choice of reflections for refinement. R-factors based on F^2^ are statistically about twice as large as those based on F, and R- factors based on ALL data will be even larger.

### Fractional atomic coordinates and isotropic or equivalent isotropic displacement parameters (Å^2^)

**Table:**

	*x*	*y*	*z*	*U*_iso_*/*U*_eq_	Occ. (<1)
P	0.40003 (3)	0.20845 (15)	0.59292 (5)	0.0338 (3)	
OW	0.5000	0.4938 (10)	0.7500	0.146 (3)	
HWA	0.5127	0.5453	0.7899	0.175*	0.50
O1	0.39553 (9)	−0.2053 (4)	0.58847 (14)	0.0526 (8)	
H1A	0.4058	−0.3239	0.6013	0.079*	
C1	0.44847 (16)	0.5766 (9)	0.3932 (3)	0.0735 (15)	
H1B	0.4461	0.5651	0.3449	0.110*	
H1C	0.4804	0.5651	0.4199	0.110*	
H1D	0.4364	0.7117	0.4020	0.110*	
O2	0.42908 (8)	0.3898 (4)	0.62967 (13)	0.0428 (7)	
C2	0.42087 (13)	0.3997 (7)	0.4134 (2)	0.0493 (10)	
C3	0.41939 (12)	0.3820 (6)	0.4812 (2)	0.0434 (9)	
H3A	0.4345	0.4833	0.5135	0.052*	
C4	0.39592 (11)	0.2167 (6)	0.50207 (18)	0.0371 (8)	
C5	0.37267 (12)	0.0663 (6)	0.45310 (19)	0.0425 (9)	
H5A	0.3567	−0.0450	0.4664	0.051*	
C6	0.37332 (13)	0.0829 (7)	0.3851 (2)	0.0485 (11)	
C7	0.39760 (14)	0.2487 (7)	0.3663 (2)	0.0538 (11)	
H7A	0.3982	0.2587	0.3206	0.065*	
C8	0.34811 (16)	−0.0813 (9)	0.3322 (2)	0.0685 (14)	
H8A	0.3523	−0.0481	0.2880	0.103*	
H8B	0.3157	−0.0793	0.3288	0.103*	
H8C	0.3605	−0.2202	0.3465	0.103*	
C9	0.28814 (17)	0.5685 (10)	0.7141 (3)	0.0817 (17)	
H9A	0.2571	0.5631	0.7178	0.123*	
H9B	0.2937	0.7059	0.6973	0.123*	
H9C	0.3097	0.5442	0.7587	0.123*	
C10	0.29420 (14)	0.3982 (7)	0.6642 (2)	0.0515 (11)	
C11	0.33647 (13)	0.3767 (6)	0.65031 (19)	0.0437 (9)	
H11A	0.3607	0.4685	0.6710	0.052*	
C12	0.34291 (12)	0.2187 (6)	0.60557 (17)	0.0350 (8)	
C13	0.30647 (12)	0.0836 (6)	0.57377 (19)	0.0405 (9)	
H13A	0.3107	−0.0214	0.5437	0.049*	
C14	0.26351 (13)	0.1041 (7)	0.5865 (2)	0.0452 (10)	
C15	0.25866 (14)	0.2611 (7)	0.6316 (2)	0.0489 (10)	
H15A	0.2302	0.2755	0.6405	0.059*	
C16	0.22411 (15)	−0.0427 (8)	0.5522 (3)	0.0649 (13)	
H16A	0.2189	−0.0389	0.5030	0.097*	
H16B	0.1965	0.0025	0.5629	0.097*	
H16C	0.2317	−0.1852	0.5687	0.097*	
C17	0.42596 (12)	−0.0481 (6)	0.62715 (19)	0.0369 (8)	
C18	0.42841 (16)	−0.0606 (7)	0.7034 (2)	0.0587 (12)	
H18A	0.4425	−0.1927	0.7222	0.088*	
H18B	0.3977	−0.0528	0.7082	0.088*	
H18C	0.4466	0.0558	0.7277	0.088*	
C19	0.47388 (13)	−0.0667 (7)	0.6165 (2)	0.0540 (11)	
H19A	0.4874	−0.2009	0.6343	0.081*	
H19B	0.4932	0.0469	0.6406	0.081*	
H19C	0.4712	−0.0577	0.5681	0.081*	

### Atomic displacement parameters (Å^2^)

**Table:**

	*U*^11^	*U*^22^	*U*^33^	*U*^12^	*U*^13^	*U*^23^
P	0.0353 (5)	0.0217 (5)	0.0407 (5)	−0.0001 (4)	0.0049 (4)	0.0001 (4)
OW	0.183 (7)	0.085 (5)	0.102 (5)	0.000	−0.065 (5)	0.000
O1	0.0576 (17)	0.0242 (13)	0.0655 (18)	−0.0009 (13)	0.0012 (14)	0.0037 (13)
C1	0.065 (3)	0.073 (3)	0.094 (4)	0.003 (3)	0.041 (3)	0.018 (3)
O2	0.0418 (14)	0.0225 (13)	0.0552 (17)	−0.0023 (12)	−0.0003 (12)	−0.0026 (12)
C2	0.042 (2)	0.049 (3)	0.061 (3)	0.009 (2)	0.023 (2)	0.012 (2)
C3	0.042 (2)	0.036 (2)	0.051 (2)	0.0029 (18)	0.0110 (18)	0.0028 (19)
C4	0.0331 (18)	0.0357 (19)	0.041 (2)	0.0043 (17)	0.0092 (15)	0.0052 (17)
C5	0.038 (2)	0.044 (2)	0.045 (2)	−0.0040 (18)	0.0094 (17)	0.0009 (19)
C6	0.042 (2)	0.060 (3)	0.042 (2)	0.006 (2)	0.0092 (18)	−0.008 (2)
C7	0.052 (2)	0.068 (3)	0.045 (2)	0.013 (2)	0.0178 (19)	0.010 (2)
C8	0.070 (3)	0.081 (4)	0.048 (3)	0.003 (3)	0.007 (2)	−0.020 (3)
C9	0.070 (3)	0.091 (4)	0.086 (4)	0.008 (3)	0.026 (3)	−0.044 (3)
C10	0.049 (2)	0.049 (3)	0.055 (3)	0.005 (2)	0.012 (2)	−0.010 (2)
C11	0.042 (2)	0.040 (2)	0.044 (2)	−0.0018 (19)	0.0036 (17)	−0.0072 (19)
C12	0.044 (2)	0.0252 (17)	0.0344 (18)	0.0025 (16)	0.0082 (15)	0.0033 (16)
C13	0.045 (2)	0.028 (2)	0.045 (2)	−0.0007 (17)	0.0090 (17)	−0.0051 (17)
C14	0.039 (2)	0.044 (2)	0.052 (2)	−0.0008 (19)	0.0119 (18)	0.002 (2)
C15	0.043 (2)	0.049 (3)	0.055 (2)	0.007 (2)	0.0163 (19)	0.002 (2)
C16	0.044 (2)	0.066 (3)	0.082 (3)	−0.012 (2)	0.013 (2)	−0.007 (3)
C17	0.0374 (19)	0.0245 (17)	0.046 (2)	0.0037 (16)	0.0073 (16)	0.0044 (16)
C18	0.076 (3)	0.045 (3)	0.053 (3)	0.011 (2)	0.016 (2)	0.011 (2)
C19	0.042 (2)	0.046 (2)	0.072 (3)	0.010 (2)	0.014 (2)	0.009 (2)

### Geometric parameters (Å, °)

**Table:**

P—O2	1.497 (2)	C9—H9A	0.9600
P—C4	1.803 (4)	C9—H9B	0.9600
P—C12	1.812 (4)	C9—H9C	0.9600
P—C17	1.837 (4)	C10—C15	1.384 (6)
OW—HWA	0.8500	C10—C11	1.387 (5)
O1—C17	1.419 (4)	C11—C12	1.393 (5)
O1—H1A	0.8200	C11—H11A	0.9300
C1—C2	1.513 (6)	C12—C13	1.390 (5)
C1—H1B	0.9600	C13—C14	1.397 (5)
C1—H1C	0.9600	C13—H13A	0.9300
C1—H1D	0.9600	C14—C15	1.378 (6)
C2—C7	1.383 (6)	C14—C16	1.505 (6)
C2—C3	1.388 (5)	C15—H15A	0.9300
C3—C4	1.389 (5)	C16—H16A	0.9600
C3—H3A	0.9300	C16—H16B	0.9600
C4—C5	1.400 (5)	C16—H16C	0.9600
C5—C6	1.383 (5)	C17—C18	1.521 (5)
C5—H5A	0.9300	C17—C19	1.524 (5)
C6—C7	1.388 (6)	C18—H18A	0.9600
C6—C8	1.521 (6)	C18—H18B	0.9600
C7—H7A	0.9300	C18—H18C	0.9600
C8—H8A	0.9600	C19—H19A	0.9600
C8—H8B	0.9600	C19—H19B	0.9600
C8—H8C	0.9600	C19—H19C	0.9600
C9—C10	1.516 (6)		
			
O2—P—C4	109.95 (17)	C15—C10—C11	118.2 (4)
O2—P—C12	110.28 (16)	C15—C10—C9	122.0 (4)
C4—P—C12	110.66 (16)	C11—C10—C9	119.8 (4)
O2—P—C17	110.91 (15)	C10—C11—C12	120.5 (4)
C4—P—C17	107.75 (18)	C10—C11—H11A	119.7
C12—P—C17	107.23 (17)	C12—C11—H11A	119.7
C17—O1—H1A	109.5	C13—C12—C11	119.7 (3)
C2—C1—H1B	109.5	C13—C12—P	124.7 (3)
C2—C1—H1C	109.5	C11—C12—P	115.6 (3)
H1B—C1—H1C	109.5	C12—C13—C14	120.6 (3)
C2—C1—H1D	109.5	C12—C13—H13A	119.7
H1B—C1—H1D	109.5	C14—C13—H13A	119.7
H1C—C1—H1D	109.5	C15—C14—C13	117.9 (4)
C7—C2—C3	118.0 (4)	C15—C14—C16	121.5 (4)
C7—C2—C1	121.8 (4)	C13—C14—C16	120.5 (4)
C3—C2—C1	120.1 (4)	C14—C15—C10	123.0 (4)
C2—C3—C4	121.5 (4)	C14—C15—H15A	118.5
C2—C3—H3A	119.2	C10—C15—H15A	118.5
C4—C3—H3A	119.2	C14—C16—H16A	109.5
C3—C4—C5	119.0 (4)	C14—C16—H16B	109.5
C3—C4—P	116.0 (3)	H16A—C16—H16B	109.5
C5—C4—P	124.9 (3)	C14—C16—H16C	109.5
C6—C5—C4	120.2 (4)	H16A—C16—H16C	109.5
C6—C5—H5A	119.9	H16B—C16—H16C	109.5
C4—C5—H5A	119.9	O1—C17—C18	110.9 (3)
C5—C6—C7	119.2 (4)	O1—C17—C19	110.9 (3)
C5—C6—C8	119.9 (4)	C18—C17—C19	111.5 (3)
C7—C6—C8	120.9 (4)	O1—C17—P	105.5 (2)
C2—C7—C6	122.0 (4)	C18—C17—P	108.5 (3)
C2—C7—H7A	119.0	C19—C17—P	109.4 (3)
C6—C7—H7A	119.0	C17—C18—H18A	109.5
C6—C8—H8A	109.5	C17—C18—H18B	109.5
C6—C8—H8B	109.5	H18A—C18—H18B	109.5
H8A—C8—H8B	109.5	C17—C18—H18C	109.5
C6—C8—H8C	109.5	H18A—C18—H18C	109.5
H8A—C8—H8C	109.5	H18B—C18—H18C	109.5
H8B—C8—H8C	109.5	C17—C19—H19A	109.5
C10—C9—H9A	109.5	C17—C19—H19B	109.5
C10—C9—H9B	109.5	H19A—C19—H19B	109.5
H9A—C9—H9B	109.5	C17—C19—H19C	109.5
C10—C9—H9C	109.5	H19A—C19—H19C	109.5
H9A—C9—H9C	109.5	H19B—C19—H19C	109.5
H9B—C9—H9C	109.5		
			
C7—C2—C3—C4	0.9 (6)	C4—P—C12—C13	52.7 (4)
C1—C2—C3—C4	−177.4 (4)	C17—P—C12—C13	−64.6 (3)
C2—C3—C4—C5	−1.0 (5)	O2—P—C12—C11	−4.9 (3)
C2—C3—C4—P	176.7 (3)	C4—P—C12—C11	−126.8 (3)
O2—P—C4—C3	0.2 (3)	C17—P—C12—C11	116.0 (3)
C12—P—C4—C3	122.3 (3)	C11—C12—C13—C14	−0.3 (5)
C17—P—C4—C3	−120.8 (3)	P—C12—C13—C14	−179.8 (3)
O2—P—C4—C5	177.7 (3)	C12—C13—C14—C15	−0.3 (6)
C12—P—C4—C5	−60.3 (4)	C12—C13—C14—C16	−179.9 (4)
C17—P—C4—C5	56.7 (3)	C13—C14—C15—C10	0.2 (6)
C3—C4—C5—C6	0.2 (5)	C16—C14—C15—C10	179.8 (4)
P—C4—C5—C6	−177.2 (3)	C11—C10—C15—C14	0.4 (7)
C4—C5—C6—C7	0.6 (6)	C9—C10—C15—C14	−179.5 (4)
C4—C5—C6—C8	179.8 (4)	O2—P—C17—O1	−179.9 (2)
C3—C2—C7—C6	−0.1 (6)	C4—P—C17—O1	−59.6 (3)
C1—C2—C7—C6	178.2 (4)	C12—P—C17—O1	59.6 (3)
C5—C6—C7—C2	−0.7 (6)	O2—P—C17—C18	61.2 (3)
C8—C6—C7—C2	−179.9 (4)	C4—P—C17—C18	−178.4 (3)
C15—C10—C11—C12	−1.0 (6)	C12—P—C17—C18	−59.2 (3)
C9—C10—C11—C12	178.8 (4)	O2—P—C17—C19	−60.6 (3)
C10—C11—C12—C13	1.0 (6)	C4—P—C17—C19	59.8 (3)
C10—C11—C12—P	−179.5 (3)	C12—P—C17—C19	178.9 (3)
O2—P—C12—C13	174.5 (3)		

### Hydrogen-bond geometry (Å, °)

**Table:**

*D*—H···*A*	*D*—H	H···*A*	*D*···*A*	*D*—H···*A*
OW—HWA···O2^i^	0.85	2.24	2.811 (3)	124
O1—H1A···O2^ii^	0.82	1.96	2.775 (4)	178
C3—H3A···O2	0.93	2.47	2.928 (5)	110
C11—H11A···O2	0.93	2.49	2.939 (5)	110

Symmetry codes: (i) −*x*+1, *y*, −*z*+3/2; (ii) *x*, *y*−1, *z*.
